# Investigation of Detection Limits and the Influence of DNA Extraction and Primer Choice on the Observed Microbial Communities in Drinking Water Samples Using 16S rRNA Gene Amplicon Sequencing

**DOI:** 10.3389/fmicb.2018.02140

**Published:** 2018-09-07

**Authors:** Jakob Brandt, Mads Albertsen

**Affiliations:** Department of Chemistry and Bioscience, Center for Microbial Communities, Aalborg University, Aalborg, Denmark

**Keywords:** drinking water, sampling, 16S rRNA gene amplicon sequencing, DNA, extraction, primer, detection limit

## Abstract

In recent years, 16S rRNA gene amplicon sequencing has been widely adopted for analyzing the microbial communities in drinking water (DW). However, no comprehensive attempts have been made to illuminate the inherent method biases specifically relating to DW communities. In this study, we investigated the impact of DNA extraction and primer choice on the observed microbial community, and furthermore estimated the detection limit of the 16S rRNA gene amplicon sequencing in these experimental settings. Of the two DNA extraction kits investigated, the PowerWater DNA Isolation Kit resulted in higher yield, better reproducibility and more OTUs identified compared to the FastDNA SPIN Kit for Soil, which is also commonly used within DW microbiome research. The use of three separate primer-sets targeting the V1-3, V3-4, and V4 region of the 16S rRNA gene revealed large differences in OTU abundances, with some of the primers unable to detect entire phyla. Estimations of the detection limit were based on bacteria-free water samples (1 L) spiked with *Escherichia coli* cells in different concentrations [10^1^–10^6^ cells/ml]. *E.* *coli* could be detected in all samples, however, samples with ∼10^1^ cells/ml had several contaminating OTUs constituting approximately 8% of the read abundances. Based on our findings, we recommend using the PowerWater DNA Isolation Kit for DNA extraction in combination with PCR amplification of the V3-4 or V4 region for DW samples if a broad overview of the microbial community is to be obtained.

## Introduction

The advent of next-generation sequencing has revolutionized the field of microbial ecology (Shokralla et al., 2012). One specific area affected by the technological advancement is drinking water (DW) where microbial identification using 16S rRNA gene amplicon sequencing holds great potential. Some of the potential applications have been discussed by Vierheilig et al. (2015) and include development of environmental molecular diagnostics, screening of bacterial communities and detection of fecal pollution in DW. However, studies have shown that numerous variables such as type of disinfectant, disinfectant concentration, temperature, pipe surface/pipe materials, nutrients levels, water age, and water flow impact the microbiome of DW (Berry et al., 2006; Ji et al., 2015; Inkinen et al., 2016). The lack of standards for the molecular work has been pointed out earlier (Vierheilig et al., 2015) and already in 2014, the need for standardization was stressed by Douterelo et al. (2014), with the expectation that standards would be developed in the near future. As of now, standards for the molecular work is still not implemented. Recently, a meta-analysis of bulk DW samples in full-scale DW distribution systems found differences in DNA extraction protocol, PCR primer choice, sequencing platform, etc. to have a stronger impact on differences between samples than type of source water and type of disinfectant (Bautista-de los Santos et al., 2016).

The applicability of 16S rRNA gene amplicon sequencing in DW is also greatly dependent on the ability of the method to detect low abundance microorganisms. Many articles have pointed out the potential use of 16S rRNA gene amplicon sequencing to detect ecologically relevant OTUs or pathogens (Berry et al., 2006; Zeng et al., 2013; Shaw et al., 2015; Bautista-de los Santos et al., 2016). However, to our knowledge, no attempts have been conducted to estimate a detection limit under conditions applied specifically for DW microbiome research.

In this study, we have taken the first steps toward developing guidelines for molecular work in relation to 16S rRNA gene amplicon sequencing of DW samples. First, we investigated the influence of two DNA extraction kits commonly used with DW [the PowerWater DNA Isolation Kit (PowerWater) (Nitzsche et al., 2015; Farenhorst et al., 2016; Fernando et al., 2016) and the FastDNA SPIN Kit for Soil (FastDNA) (Li et al., 2010; Huang et al., 2014; Belila et al., 2016)]. Then we investigated the impact of the choice of primers by using primer-sets targeting the V1-3, V3-4, and the V4 region of the 16S rRNA gene, which all have been used in previous DW studies (Huang et al., 2014; Prest et al., 2014; Bautista-de los Santos et al., 2015; El-Chakhtoura et al., 2015; Gomez-Alvarez et al., 2015; Farenhorst et al., 2016; Liu et al., 2016). Finally, the detection limit of the employed 16S rRNA gene amplicon approach was estimated based on spike-in experiments using *Escherichia coli* (*E. coli*) cells in concentrations spanning 10^1^–10^6^ cells/ml.

## Materials and Methods

### Drinking Water Sample Location

All DW samples were collected from a regular tap in the lab of an office building located in Aalborg East (57.014598 °N, 9.984849 °E). The water originated from a spring approximately 2 km south of the building (56.997748 °N, 9.965075 °E). The ground water entered the DW distribution system without any kind of treatment.

### Sample Collection of Drinking Water Samples

All DW samples were collected in accordance with the manual “Drikkevand. Manual for prøvetagning” composed by the reference laboratory of the Danish Nature Agency (Naturstyrelsens Referencelaboratorium, 2015). In short, the sampling site was inspected prior to sample collection in order to prevent contamination from the surroundings. Attachments were disassembled and impurities such as limescale and grease were removed. The tap was turned completely on and off repeatedly to wash away impurities and subsequently immersed in 99% ethanol for at least 2 min. The water was left running at a steady flow for at least 5 min prior to sampling. All samples were collected in 1 L sterile, disposable PE bottles (Corning Life Science).

Isolation of bacteria cells was carried out by a filtration step immediately after the sampling procedure. A filtration setup (Sartorius) consisting of a Microsart^®^ e.jet vacuum pump, a Combisart^®^ 3-branch Manifold and disposable Microsart^®^ 250 ml funnels were used. Bacterial biomass was collected on 0.2 μm pore size cellulose nitrate filters (Sartorius). Negative controls were included containing nuclease-free water (QIAGEN). After filtration, the filters were stored at -20°C prior to DNA extraction.

### DNA Extraction From Drinking Water Samples

Isolation of DNA was performed with the PowerWater DNA Isolation Kit (now known as DNeasy PowerWater Kit, QIAGEN) or the FastDNA SPIN Kit for Soil from (MP Biomedicals). The PowerWater DNA Isolation Kit was used following the recommended protocol by the manufacturer with only one adjustment. The isolated DNA was eluted in a final volume of 60 μl. The FastDNA SPIN Kit for Soil was used with two modifications to the recommended protocol. The input material was the 0.2 μm filters cut into pieces, and the bead beating step consisted of 4 s × 40 s at 6 m/s on a FastPrep-24 instrument. After the DNA extraction step, all samples were stored at -20°C prior to preparation of sequencing libraries. DNA concentrations were measured fluorometrically with Quant-iT HS DNA Assay (Thermo Fisher Scientific) on either an Infinite M1000 PRO (Tecan) or a Qubit^®^ Fluorometer (Thermo Fisher Scientific).

### Sample Preparation and Sequencing of 16S rRNA Gene Libraries

Samples prepared for sequencing of the V1-3 variable region of the 16S rRNA gene were conducted as described by Albertsen et al. (2015). PCR reactions were run with 2 μl extracted DNA as template in the PCR reactions (25 μl) which also contained dNTPs (400 nM in total), MgSO_4_ (1.5 mM), Platinum^®^ Taq DNA polymerase High Fidelity (1U), 1X Platinum High Fidelity buffer (Thermo Fisher Scientific), as well as bar-coded library adaptors [27F AGAGTTTGATCCTGGCTCAG and 534R ATTACCGCGGCTGCTGG (Human Microbiome Project Consortium, 2012)] (400 nM). Thermocycler settings for V1-3 PCR: initial denaturation at 95°C for 2 min, 30 cycles of 95°C for 20 s, 56°C for 30 s, 72°C for 60 s and final elongation at 72°C for 5 min. A negative and positive control containing nuclease-free water and isolated *E. coli*-DNA, respectively, were included in all PCR amplification steps. All PCR steps were performed in duplicates and pooled after amplification.

Preparation of samples for sequencing of the V3-4 region [341F CCTAYGGGRBGCASCAG and 806R GGACTACNNGGGTATCTAAT (Sundberg et al., 2013)] and the V4 region [A519F CAGCMGCCGCGGTAA and S-D-Bact-0785-b-A-18 TACNVGGGTATCTAATCC (Klindworth et al., 2013)] occurred with the same procedure, but using a two-step PCR-amplification. Thermocycler settings for the first amplicon PCR: initial denaturation at 95°C for 2 min, 35 cycles of 95°C for 20 s, 50°C for 30 s, 72°C for 60 s and final elongation at 72°C for 5 min. The second library PCR was run with 2 μl cleaned amplicon PCR product as template in the PCR reactions (25) which also contained X5 PCRBIO reaction buffer (x1) and PCRBIO Hifi polymerase (1U). Thermocycler settings for the library PCR: initial denaturation at 95°C for 2 min, 8 cycles of 95°C for 20 s, 55°C for 30 s, 72°C for 60 s and final elongation at 72°C for 5 min. The library PCR was performed in single reactions.

The V3-4 primer-set was used for the comparison of extraction methods and the detection limit experiment.

Amplicon libraries were purified using Agencourt AMpure XP bead (Beckmann Coulter). A sample:bead solution ratio of 5:4 was used, and the purified DNA was eluted in nuclease-free water. Library concentrations were measured with Quant-iT HS DNA Assay (Thermo Fisher Scientific) and the purified amplicon products were visualized on a TapeStation 2200 using D1K ScreenTapes (Agilent). All samples were pooled into one tube in equimolar concentrations. Sequencing of the library pools was carried out on a MiSeq (Illumina).

### Detection Limit Experiment

Estimation of the lower detection limit for 16S rRNA gene sequencing was based on sequencing data from a dilution series consisting of DNA- and bacteria-free water spiked with *E. coli* cells. In order to ensure no presence of other bacterial species in the water, approximately 20 L of DW was autoclaved. Subsequently, 1 ml DEPC (Sigma-Aldrich) per liter water was added and incubated for at least 16 h at room temperature to break down any remaining DNA-strands. A final autoclaving step was performed to inactivate the DEPC molecules. The *E. coli* cells were grown from a pure culture (DSMZ 30083) in a lysogeny broth medium to a final OD_600_ = 0.14. Using DAPI staining, the bacterial concentration of the *E. coli* suspension was calculated to 9.8 × 10^7^ cells/ml. Immediately after the desired OD was reached, 50 ml *E. coli*-suspension was transferred to 900 ml sterile water (4°C) to inhibit further growth. The 10-fold dilution series was made in triplicates with concentrations ranging from ∼10^6^ to ∼10^1^ cells/ml. Within 2 h after the dilution series were prepared, all samples were collected with the filtration step described above. The DNA extraction step was performed with the PowerWater DNA Isolation Kit.

### Bioinformatics – Processing of Amplicon Sequences

Initially, a quality trimming of forward and reverse reads was conducted using Trimmomatic v. 0.32 (Bolger et al., 2014) with the settings SLIDINGWINDOW: 5:3 and MINLEN: 275 (sequences generated with the V4 primer had MINLEN: 225). The trimmed forward and reverse reads were merged using FLASH v. 1.2.7 (Magoc and Salzberg, 2011) with the settings -m 25 -M 200 (settings for V4 samples: -m 10 -M 250). All merged reads were screened for any potential PhiX contamination using USEARCH version 7 (Edgar, 2010). PhiX is a small virus genome serving as a positive control in sequencing runs. The reads were dereplicated and formatted for use in the UPARSE workflow (Edgar, 2013). The dereplicated reads were then clustered using the USEARCH7 command -cluster_otus with default settings. OTU abundances were estimated using USEARCH7 with the -usearch_global command and the following options: -id 0.97 -maxaccepts 0 -maxrejects 0. Taxonomic classification was based on the Ribosomal Database Project (RDP) Classifier (Wang et al., 2007) with the MiDAS_S123 database (McIlroy et al., 2015), which is a curated database based on the SILVA database, release 123 (Quast et al., 2013). The classification was performed using the QIIME script *parallel_assign_taxonomy_rdp.py* with the minimum confidence set to 0.8 (Caporaso et al., 2010). All sequencing data was processed in R using the package ampvis2 (Andersen et al., 2018) for visualization of amplicon sequencing data.

## Results

To elucidate the extent to which methodology contributes to variation between studies, we designed experiments focusing on two of the key steps during the lab work: DNA extraction and PCR amplification. The test of extraction methods was primarily based on parameters such as yield, number of observed OTUs and variation between replicates. The experiment revealed large discrepancies between the two extraction methods for all three parameters. The primer test was conducted to illuminate which primer set that covered the broadest range of microbial diversity in DW. Equivalent to the extraction method test, large differences in the microbial community was observed based on primer choice. However, the available literature concerning 16S rRNA gene amplicon sequencing of DW samples shows that a wide range of methods have been applied (**Table 1**).

**Table 1:** Literature survey of 12 articles published in recent years relating to DW.

	Reference	Sampling method	Sampling volume [L]	Extraction method	Primer target region	PCR cycles
1	Hull et al., 2017	Filtration (0.2 mm pore-size)	1.5–2	Phenol–chloroform method	V1-2	26^∗^
2	Farenhorst et al., 2016	Filtration (0.2 mm pore-size)	0.5	PowerWater DNA Isolation Kit	V4	35
3	Liu et al., 2016	Filtration (0.2 mm pore-size)	100	FastDNA SPIN Kit	V4	30
4	Belila et al., 2016	Filtration (0.2 mm pore-size)	3	FastDNA SPIN Kit for Soil	V4-5	30
5	Bautista-de los Santos et al., 2015	Filtration (0.2 mm pore-size)	13–17	Phenol-chloroform method	V4	30
6	Gomez-Alvarez et al., 2015	Filtration (0.2 mm pore-size)	5	UltraClean Soil DNA Kit	V1-3	35
7	Roeselers et al., 2015	Filtration (0.2 mm pore-size)	1	Lysis buffer and beat beating	V5-6	45
8	El-Chakhtoura et al., 2015	Filtration (0.2 mm pore-size)	2	FastDNA SPIN Kit	V3-4	28
9	Shaw et al., 2015	Centrifugation	0.05	UltraClean Soil DNA Kit	V3	30
10	Prest et al., 2014	Filtration (0.2 mm pore-size)	2	FastDNA SPIN Kit	V3-4	30
11	Holinger et al., 2014	Filtration (0.2 mm pore-size)	1.5	Lysis buffer and bead beating	V1-2	30
12	Huang et al., 2014	Water purifiers	1000	FastDNA Soil Kit	V3-4	30

*^∗^The exact number of PCR cycles used in the article was unclear.*

Apart from choice of sampling method, hardly any consensus is observed for any of the steps listed in **Table 1**.

### Comparison of DNA Extraction Kits

The general performance of the PowerWater and FastDNA DNA extraction kits were evaluated using 10 biological replicates of 2 L DW with five replicates for each kit (**Table 2**). The PowerWater kit had measurable DNA concentrations after extraction for all replicates with an average DNA concentration of 0.33 ng/μl. Conversely, none of the replicates extracted with the FastDNA kit had measurable DNA concentrations (limit of quantification 0.02 ng/ul). However, despite low DNA yields all samples extracted using the FastDNA kit also produced useful sequencing libraries.

**Table 2:** Overview of metadata and raw data from the extraction kit comparison test.

Sample	Extraction kit	Volume (L)	Replicate	Extraction DNA concentration [ng/μl]	Library DNA concentration [ng/μl]	Observed OTUs	Number of reads
1	FastDNA	2	a	BDL	3.4	799	43,699
2	FastDNA	2	b	BDL	2.7	616	44,625
3	FastDNA	2	c	BDL	2.1	691	22,215
4	FastDNA	2	d	BDL	4	810	43,797
5	FastDNA	2	e	BDL	2.5	598	45,530
6	FastDNA	–	Blank	BDL	BDL	9	354
7	PowerWater	2	a	0.2	28.3	1,368	36,823
8	PowerWater	2	b	0.3	27.3	1,378	39,829
9	PowerWater	2	c	0.4	24.2	1,374	38,121
10	PowerWater	2	d	0.4	35.6	1,407	34,372
11	PowerWater	2	e	0.3	27.8	1,233	40,082
12	PowerWater	–	Blank	BDL	BDL	41	1,940
13	PCR positive control	–	–	–	31.0	4	47,317
14	PCR negative control	–	–	–	BDL	3	177

*The table includes five replicate samples extracted for each of the two different extraction kits as well as a blank extraction for each kit and PCR controls. Library DNA concentration refers to the concentration after the final PCR step (the V3-4 region was targeted). Notice that the number of different OTUs observed for each sample is based on a subset of reads (normalized to 20,000 reads for each sample). The last column lists the total number of processed reads for each sample. BDL, below detection limit.*

**FIGURE 1:**
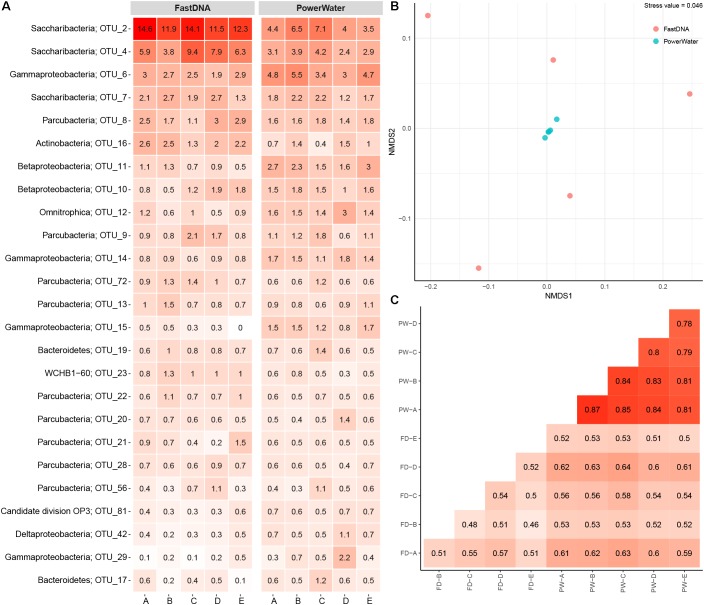
**(A)** Heatmap of 2 L DW samples extracted with two different kits. Each column represents a sample and is grouped by extraction kit. The rows list the 25 most abundant OTUs across the samples. Each OTU is assigned with its phylum classification. The numbers state the relative read abundance. **(B)** Ordination by non-metric multidimensional scaling based on Bray–Curtis dissimilarity. Each sample is represented as a dot and is colored based on extraction method. **(C)** Sample-by-sample comparison of the 10 replicates (FD, FastDNA; PW, PowerWater). The similarity between any two samples are displayed as a percent from 0 to 1. The numbers are based on Bray–Curtis measures.

The number of observed OTUs ranged from ∼600 to ∼800 for the FastDNA samples, whereas the PowerWater samples had between ∼1200 and ∼1400 observed OTUs (normalized to 20,000 reads). Applying a Student’s *t*-test, the PowerWater samples had a significantly higher number of OTUs compared to the FastDNA samples (*p*-value = 2.1 × 10^−6^, *n* = 10). Only focusing on the 25 most abundant OTUs from the samples, as visualized in **Figure 1A**, revealed that the largest differences in abundances between the two kits are associated to the Saccharibacteria OTUs. However, a clearer distinction of the two extraction kits emerged by using non-metric multidimensional scaling to visualize the data as seen in **Figure 1B**. A stark contrast between the two extraction kits was seen. The PowerWater replicates form a distinct cluster whereas the FastDNA replicates are scattered around the PowerWater replicates. The trend observed in the ordination plot is further emphasized in **Figure 1C**. Here, the beta-diversity is illustrated in a sample-to-sample manner, and a clear separation between the two extraction kits can be observed. The PowerWater kit demonstrated better reproducibility as the similarity scores for the five replicates ranged from 0.78 to 0.87 (where a value of one represents two samples with identical microbial composition). Conversely, similarity scores for the FastDNA kit ranged from 0.46 to 0.57.

### Comparison of Primer-Sets

**FIGURE 2:**
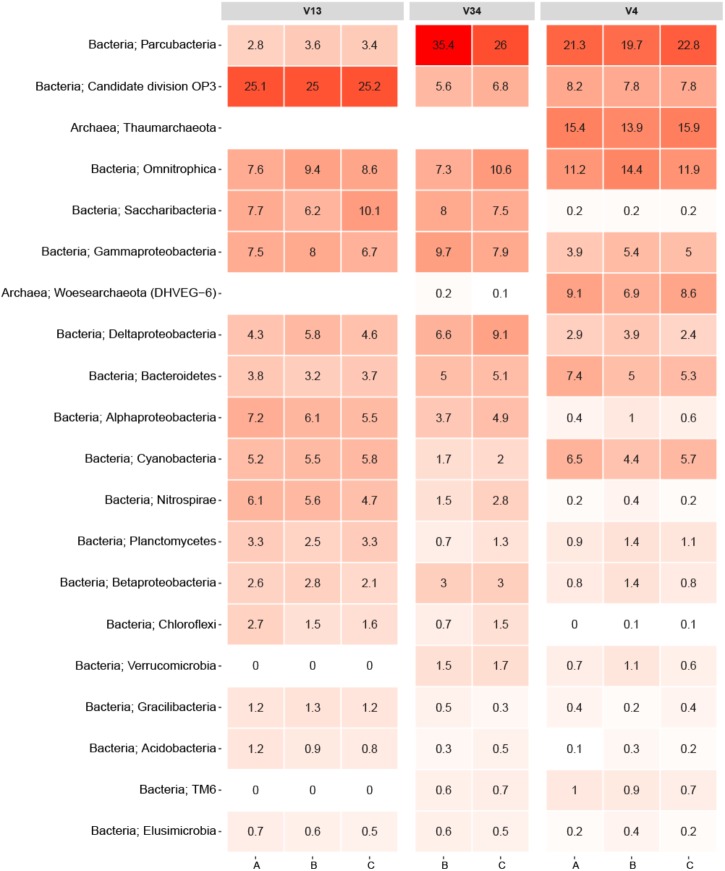
Heatmap of the primer-set comparison. Each column represents a sample denoted by its replicate and grouped by the variable region of the 16S rRNA gene targeted. The rows list the 20 most abundant phyla across the samples. Each phylum is assigned with its kingdom. The numbers state the relative read abundance.

To facilitate direct comparisons between different primer-sets, an experiment was performed where DNA was extracted with the PowerWater kit from three 2 L biological replicate samples of DW. Each replicate DNA sample was PCR amplified using three different primer-sets targeting the V1-3, V3-4, and V4 variable region of the 16S rRNA gene. An overview of the raw data can be found in **Supplementary Table 1**. While the V34 primer-set produced fewer OTUs than the other primer-sets (normalized to 10,000 reads), the largest difference between the primer-sets was observed in the varying abundances of the 20 most abundant phyla displayed in **Figure 2**. Note, replicate A from the V3-4 primer-set failed to generate reads during the sequencing and is omitted from **Figure 2**.

### Estimating the Detection Limit of 16S rRNA Gene Amplicon Sequencing

A detection limit experiment was designed comprising of 21 L autoclaved and DEPC-treated DW samples of 1 L each. The samples exclusively contained *E. coli* cells in varying concentrations with a bacterial concentration ranging from ∼10^6^ to 10^1^ cells/ml. This covered the interval typically associated with DW between 10^3^ and 10^5^ cells/ml (Pinto et al., 2012).

**FIGURE 3:**
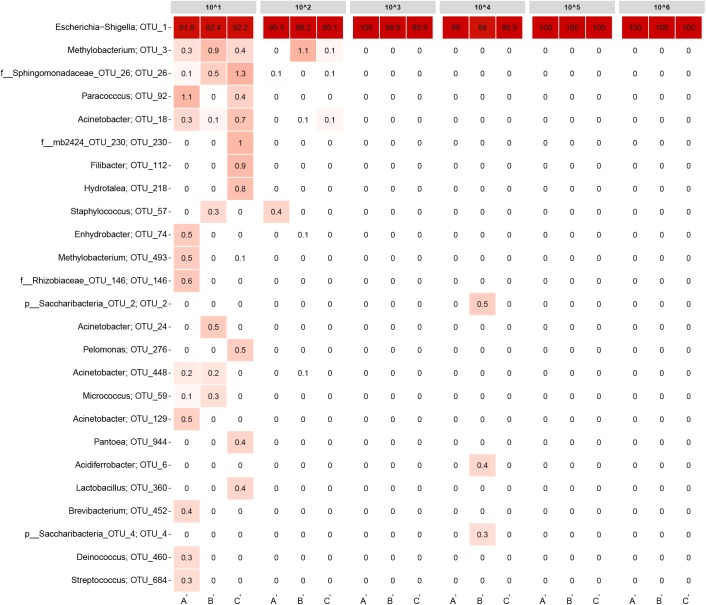
Heatmap of the detection limit experiment. Each column represents a sample and is grouped by bacteria concentration. The rows list the 25 most abundant OTUs across the samples. Each OTU is assigned with its genus or the closest possible taxonomic rank. The numbers state the relative read abundance.

Ideally, all samples should only have contained *E. coli* cells. Hence, any other OTUs detected need to be considered as contamination from the workflow (normalized to 20,000 reads). Samples with bacterial concentrations equivalent to normal DW or higher almost exclusively contained *E. coli* (**Figure 3**; OTU_1), disregarding replicate B from the ∼10^4^ cells/ml sample. More interestingly, also some of the low biomass samples had read abundances for *E. coli* above 90%. For the triplicates containing ∼10^2^ cells/ml, approximately 1–2% of the read abundances could be attributed to contamination shared among only a few OTUs. For the ∼10^1^ cells/ml samples, several contaminating OTUs were detected constituting approximately 8% of the read abundances. An overview of the raw data is listed in **Supplementary Table 2**.

The detection limit experiment also highlights the importance in including appropriate control samples in the workflow and why the read abundances generated from 16S rRNA gene amplicon sequencing should be evaluated in combination with extraction and library concentrations from the lab work. In this experiment, the most diluted samples in particular need to be assessed if they have produced reliable data. For this experiment, three control samples of ∼1 L each were included that did not contain *E. coli*, a blank sample was included for the DNA extraction step (only containing extraction buffer) and a positive and negative control was included in the PCR. In **Supplementary Figure 2**, a heatmap of the detection limit experiment is displayed including the controls samples and the blank extraction. All the controls contained *E. coli* in varying abundances (9.9–76.7%), most likely originating from crossflow contamination during the workflow. However, since all the control samples had very low library concentrations (≤1.5 ng/μl), the read abundances connected to the read abundances may easily be distorted. For all the library concentrations listed in **Supplementary Table 2**, only replicate C from the 10^1^ sample had a library concentration in the same range as the control samples and should be disregarded in the analysis.

## Discussion

The DNA extraction step has been pointed to as a highly critical step in sequencing-based analyses (Albertsen et al., 2015; Vierheilig et al., 2015). Our DNA extraction kit comparison demonstrated significant differences in performance between the two kits used. Most notably, the amount of DNA isolated from the DW samples differed by at least one order of magnitude. For samples containing low microbial biomass in particular, the DNA yield is extremely important as low amounts of starting material may be effectively swamped by contaminating DNA (Salter et al., 2014). This scenario was clearly illustrated by the several contaminants detected in the 10^1^ cells/ml samples from the detection limit experiment. As a consequence of the much higher DNA yield from samples extracted with the PowerWater kit, these samples are less vulnerable to contamination. More often than not, 16S rRNA gene amplicon studies relating to DW omit raw experimental data like DNA extraction concentrations and library concentrations. This limits the possibility of assessing the quality of the sequencing results.

Despite all libraries were normalized prior to sequencing, the number of reads ranged from approximately 22,000 to 46,000 (disregarding extraction blanks and PCR controls). This variation can be attributed to more than one factor. Naturally, the quality of the individual library plays a significant role as libraries with low DNA concentrations (<1 ng/μl) often fail due to too low input material. However, the variation seen between libraries of high quality are predominantly caused by chance. The assay kit used for quantification prior to normalization has an accuracy within 15% (Invitrogen, 2016). Combine this with the pipetting accuracies when handling small volumes (ISO 8655 certified pipettes have a permissible random error of ±5% for 1 μl) and the number of reads obtained after sequencing will display variation.

The FastDNA kit underestimated the microbial diversity in DW samples as the samples extracted with the PowerWater kit had a significantly higher number of OTUs observed. However, the large number of OTUs only observed in the PowerWater samples were low abundance OTUs as all of the 25 most abundant OTUs were observed in all samples regardless of extraction method, see **Figure 1A**.

Even though the comparison was based on five biological replicates for each extraction kit, some degree of variability between the replicates was present. The FastDNA samples in particular proved to be relatively dissimilar (**Figure 1B**), but also from the heatmap (**Figure 1A**) relatively large differences in OTU abundances between biological replicates were observed. Variability is a consequence of environmental heterogeneity and also introduced during sampling, extraction and sequencing (Prosser, 2010). First of all samples should be collected that are representative, i.e., a sample in which the measured parameter (the microbial community in this case) is the same in the sample as in source from which the sample was collected (Erickson et al., 2013). In general, samples only remain representative for a short period of time and at a specific location (Erickson et al., 2013). Despite studies have demonstrated that the core microbial community in DW is rather stable over time (El-Chakhtoura et al., 2015; Roeselers et al., 2015), different microbial communities within one building have been reported earlier (Inkinen et al., 2016). In this experiment, environmental heterogeneity does not seem to be the largest factor as the internal variance is larger for the FastDNA kit compared to the PowerWater kit, which had identical sampling schemes (**Figure 1B**). However, we also have to state, once again, that biological replication should always be carried out (Prosser, 2010).

A large source of variation is the DNA extraction kit, as has been demonstrated in several studies (Albertsen et al., 2015; Vierheilig et al., 2015). However, another interlinked source of variation is low template concentrations in the PCR, which have been shown to cause significant alterations in the observed microbial communities at concentrations (Multinu et al., 2018). Hence, the larger variations observed using the FastDNA kit might be attributed to the low concentrations of DNA obtained (below detection limit; <0.2 ng/ul).

On a side note, only four of the OTUs in **Figure 1A** were assigned with their genus classification (see **Supplementary Figure 1** for heatmap with genus classification) underlining the need for more comprehensive databases of 16S rRNA gene sequences in DW. From a historical point of view, the DW microbiome has been difficult to characterize as estimations point to only approximately 0.25% of microorganisms in DW are cultivable (Roeselers et al., 2015). Despite the fact that taxonomy based on the 16S rRNA gene is currently the most widely applied method in microbiology, numerous microorganisms still belong to taxa that have not yet been characterized (McDonald et al., 2012). Other studies have pointed to DW as ecosystems potentially inhabiting a vast amount of undiscovered bacterial diversity (Luef et al., 2015; Bruno et al., 2017). However, a better taxonomic classification should be obtained concurrently as new methods are making it possible to expand the reference databases by orders of magnitude at relatively little cost and time (Karst et al., 2018).

As expected, the sequencing data proved to be consistent with the literature as it showed that primer selection had a significant influence on the observed bacterial community (Albertsen et al., 2015). In particular, the V1-3 primer differed from the V3-4 and V4 primers owing to its inability to amplify PCR products from the archaeal phyla. An *in silico* analysis of the V1-3 primer specificity using TestPrime by Klindworth et al. (2012) revealed that the primer-set was not designed to target archaea, which explains the read abundances of 0% for Thaumarchaeota and Woesearchaeota. Conversely, the V4 primer samples had both of the archaeal phyla as some of the most abundant. Moreover, the V1-3 primer also seemed to either underestimate or completely miss species from the phylum Verrucomicrobia and TM6. This was not the case for samples amplified with the two other primers as all these samples had read abundances of at least 0.6% for these phyla. This is consistent with findings in a recent study by Zhang et al. (2017), which also indicated that some primer-sets are incapable of detecting specific phyla. The study by Zhang et al. (2017) investigated the microbial profiles of DW using primer-sets targeting the V3, V4, and the V6 region. It should also be emphasized that the results from the primer test do not give a complete understanding of the primer bias related to DW as any phyla absent from the samples would not be detectable.

Another notable difference was the phylum Parcubacteria, which had an average read abundance of 3.3% for the V1-3 primer samples compared to average read abundances of 30.7 and 21.3% for the V3-4 and V4 primer samples, respectively (**Figure 2**). Despite the fact that the V4 primer proved to be better suited for detection of certain archaeal phyla, it also demonstrated an underestimation of other phyla such as Saccharibacteria, Nitrospirae, Chloroflexi, and Acidobacteria compared to the other primers.

Recently, Bautista-de los Santos et al. (2016) published an article in which they meta-analyzed microbial communities in full scale DW distribution systems based on 21 studies spread across seven countries around the world. A comparison between the top 20 phyla in **Figure 2** and the occurrence of main bacterial phyla reported in the meta-analysis revealed that 18 of the 20 phyla overlapped including the two archaeal phyla. Only OP3, TM6, Gracilibacteria, and Woesearchaeota were not observed in the meta-analysis. The samples analyzed in the study by Bautista-de los Santos et al. (2016) originated from both disinfectant treated environments as well as disinfectant residual-free environments. Conversely, samples from this paper originated exclusively from DW not treated with any disinfectants, and therefore the comparison was only related to disinfectant residual-free DW.

Overall, the primer test documented that primers targeting different variable regions on the 16S rRNA gene introduces significant biases to DW samples. Notwithstanding the fact that only three different primer-sets were tested, similar results should be expected regardless of the variable regions being targeted (also illustrated by the study from Zhang et al., 2017). The results obtained furthermore emphasize the futility in comparing specific datasets applying different primers for the PCR step. Ideally, a consensus for a primer set targeting a specific variable region should be attained, although the optimal primer choice would greatly dependent on the focus of the research. As **Figure 2** demonstrates, the three primers used in the test each detected specific phyla better than the two others. For example, spp. from the Nitrospirae phylum would be of particular interest in connection to DW distribution systems utilizing chloramines as a disinfectant. This owes to the release of ammonia during chloramine decay which can result in nitrification by which ammonia is converted to nitrite and nitrate (Zhang et al., 2009). If an analysis of a DW distribution system was conducted with specific focus on nitrification, the V1-3 primer would be the most suited option for detecting Nitrospirae OTUs. However, in our study the primer-set targeting the V3-4 and V4 region did generally display the best ability to capture the overall microbial diversity in the DW samples compared to the V1-3. In our evaluation of the three primer-sets it should be noted that the primer-sets ability to identify different bacteria was valued higher than accurate OTU abundances. This owes to the fact that identification of bacteria remains the main goal of the method. Also, abundances may be misleading due to copy number bias (Kembel et al., 2012). Sequencing of a mock community would be an effective strategy to address the question of which primer-set that results in the most accurate profile of the microbial community. However, constructing a representative mock community of the DW microbiome would be challenging.

Sequencing results from the detection limit experiment illustrated that 16S rRNA gene sequencing can detect bacteria species in relatively low concentrations, given proper care to avoid contamination. Only for the most diluted samples, numerous contaminating OTUs were detected. It is plausible that *E. coli* would be detected even if an additional 10-fold dilution of the ∼10^1^ cells/ml samples were made. Based on the calculations from the dilution series, the ∼10^1^ cells/ml replicates account for roughly 50,000 *E. coli* cells in total. However, if more diluted samples were included in the experiment, the number of target cells would eventually be lost in contaminating DNA. Hence, the limiting factor for detecting low abundance bacteria is most likely the presence of contamination rather than the method’s ability to successfully extract and amplify DNA from target organisms. Limiting the amount of contamination may prove to be difficult, as findings by Salter et al. (2014) have previously shown a plethora of contaminating genera to be present in extraction kits and lab reagents commonly used for 16S rRNA gene sequencing. The majority of the non-*Escherichia–Shigella* genera observed in **Figure 3** were also reported by Salter et al. (2014) as contaminants. Some of the genera were represented by more than one OTU.

The detection limit experiment demonstrated that the workflow used was very effective when focusing on the core microbial community from samples with bacterial concentrations in the range of normal DW (10^3^ to 10^5^ cells/ml). For almost all of the samples, contamination was of no concern. Only replicate B from the ∼10^4^ cells/ml sample had notable read abundances for non-*E. coli* OTUs combined with the lowest *E. coli* read abundance for all samples (88%). Still, the contamination observed in **Figure 3** was not overlapping with the contaminating OTUs for the ∼10^1^ and ∼10^2^ cells/ml samples.

## Conclusion

The field of 16S rRNA gene sequencing analysis of DW needs methodological standardization if results are to be compared across studies. We recommend the use of PowerWater DNA Isolation Kit for DNA extraction of bulk DW samples and PCR amplification of the V3-4 or V4 variable region of the 16S rRNA gene. Furthermore, we encourage researchers to include raw experimental data such as extraction and library concentrations in research being published. Finally, biological replicates and negative controls should always be included in order to assess data variability and contamination.

## Author Contributions

JB and MA designed the experiment, analyzed the data, and wrote the manuscript. JB performed all laboratory-related work.

## Conflict of Interest Statement

MA is co-founder of the DNA sequencing and analysis company DNASense ApS. The remaining author declares that the research was conducted in the absence of any commercial or financial relationships that could be construed as a potential conflict of interest.
