# Supplementary material for: Investigation of Detection Limits and the Influence of DNA Extraction and Primer Choice on the Observed Microbial Communities in Drinking Water Samples Using 16S rRNA Gene Amplicon Sequencing
Source: Front Microbiol. 2018 Sep 7;9:2140. doi: 10.3389/fmicb.2018.02140 (PMC6137089; doi:10.3389/fmicb.2018.02140)
Supplement: Supplementary file 1 [file Data_Sheet_1.PDF]

*Supplementary Material*

# Investigation of Detection Limits and the Influence of DNA Extraction and Primer Choice on the Observed Microbial Communities in Drinking Water Samples using 16S rRNA Gene Amplicon Sequencing

**Jakob Brandt[1], Mads Albertsen[1]***

*** Correspondence:** Mads Albertsen: ma@bio.aau.dk

## 1 Supplementary Figures and Tables

**Supplementary table 1.** Overview of metadata and raw data from the primer-set test. The table includes three samples for each primer-set excluding PCR controls. Library DNA concentration refers to the concentration after the final PCR step. Notice that the number of different OTUs observed for each sample is based on a subset of reads (normalized to 10,000 reads for each sample). The last column lists the total number of processed reads for each sample.

| Sample | Primer-set | Replicate | Extraction DNA concentration [ng/µl] | Library DNA concentration [ng/µl] | Observed OTUs | Number of reads |
|---|---|---|---|---|---|---|
| 1 | V13 | a | 2.34 | 23.4 | 865 | 10,122 |
| 2 | V13 | b | 1.63 | 20.9 | 893 | 26,005 |
| 3 | V13 | c | 1.37 | 21.2 | 850 | 11,890 |
| 4 | V34 | a | 2.34 | 18.9 | 0 | 0 |
| 5 | V34 | b | 1.63 | 17.6 | 342 | 21,724 |
| 6 | V34 | c | 1.37 | 21 | 342 | 17,160 |
| 7 | V4 | a | 2.34 | 21.2 | 852 | 50,165 |
| 8 | V4 | b | 1.63 | 19.1 | 875 | 62,417 |
| 9 | V4 | c | 1.37 | 18.6 | 876 | 48,136 |
| 10 | V13 PCR neg | - | - | 2.89 | 51 | 697 |
| 11 | V13 PCR pos | - | - | 29.7 | 20 | 19,523 |
| 12 | V34 PCR neg | - | - | 0.148 | 0 | 0 |
| 13 | V34 PCR pos | - | - | 29.6 | 993 | 48102 |
| 14 | V4 PCR neg | - | - | 0.093 | 0 | 0 |
| 15 | V4 PCR pos | - | - | 31.7 | 6 | 2260 |

**Supplementary table 2.** Overview of metadata and raw data from the detection limit experiment. The table includes all samples from the dilution series as well as control samples. Library DNA concentration refers to the concentration after the final PCR step (the V1-3 region was targeted). The last column lists the total number of processed reads for each sample. Notice that the number of different OTUs observed for each sample is based on a subset of reads (normalized to 20,000 reads for each sample). UD = under detection limit.

| Sample | Cell concentration [cells/ml] | Replicate | Extraction DNA concentration [ng/µl] | Library DNA concentration [ng/µl] | Observed OTUs | Number of reads |
|---|---|---|---|---|---|---|
| 1 | $10^6$ | a | 13.8 | 27.1 | 4 | 47,863 |
| 2 | $10^6$ | b | 21.2 | 27.9 | 3 | 47,476 |
| 3 | $10^6$ | c | 15.8 | 14.6 | 2 | 46,632 |
| 4 | $10^5$ | a | 13 | 12.4 | 2 | 42,906 |
| 5 | $10^5$ | b | 19.8 | 24.9 | 6 | 47,822 |
| 6 | $10^5$ | c | 17.6 | 24.9 | 2 | 47,493 |
| 7 | $10^4$ | a | 0.6 | 17.8 | 131 | 47,642 |
| 8 | $10^4$ | b | 0.6 | 28.1 | 660 | 45,679 |
| 9 | $10^4$ | c | 0.3 | 8.9 | 19 | 42,944 |
| 10 | $10^3$ | a | UD | 16.9 | 8 | 47,960 |
| 11 | $10^3$ | b | UD | 9.7 | 7 | 45,608 |
| 12 | $10^3$ | c | UD | 9 | 7 | 48,060 |
| 13 | $10^2$ | a | UD | 4.6 | 10 | 46,200 |
| 14 | $10^2$ | b | UD | 23.9 | 26 | 47,963 |
| 15 | $10^2$ | c | UD | 24.3 | 25 | 48,364 |
| 16 | $10^1$ | a | UD | 4.2 | 35 | 48,005 |
| 17 | $10^1$ | b | UD | 8.8 | 59 | 47,986 |
| 18 | $10^1$ | c | UD | 1 | 35 | 37,997 |
| 19 | control | a | UD | 1.5 | 50 | 29,131 |
| 20 | control | b | UD | 1.2 | 32 | 34,893 |
| 21 | control | c | UD | 1.2 | 48 | 40,993 |
| 22 | control | Extraction blank | - | 0.4 | 46 | 18,384 |
| 23 | control | PCR neg | - | UD | 13 | 1,238 |
| 24 | control | PCR pos | - | 12.1 | 2 | 47,947 |

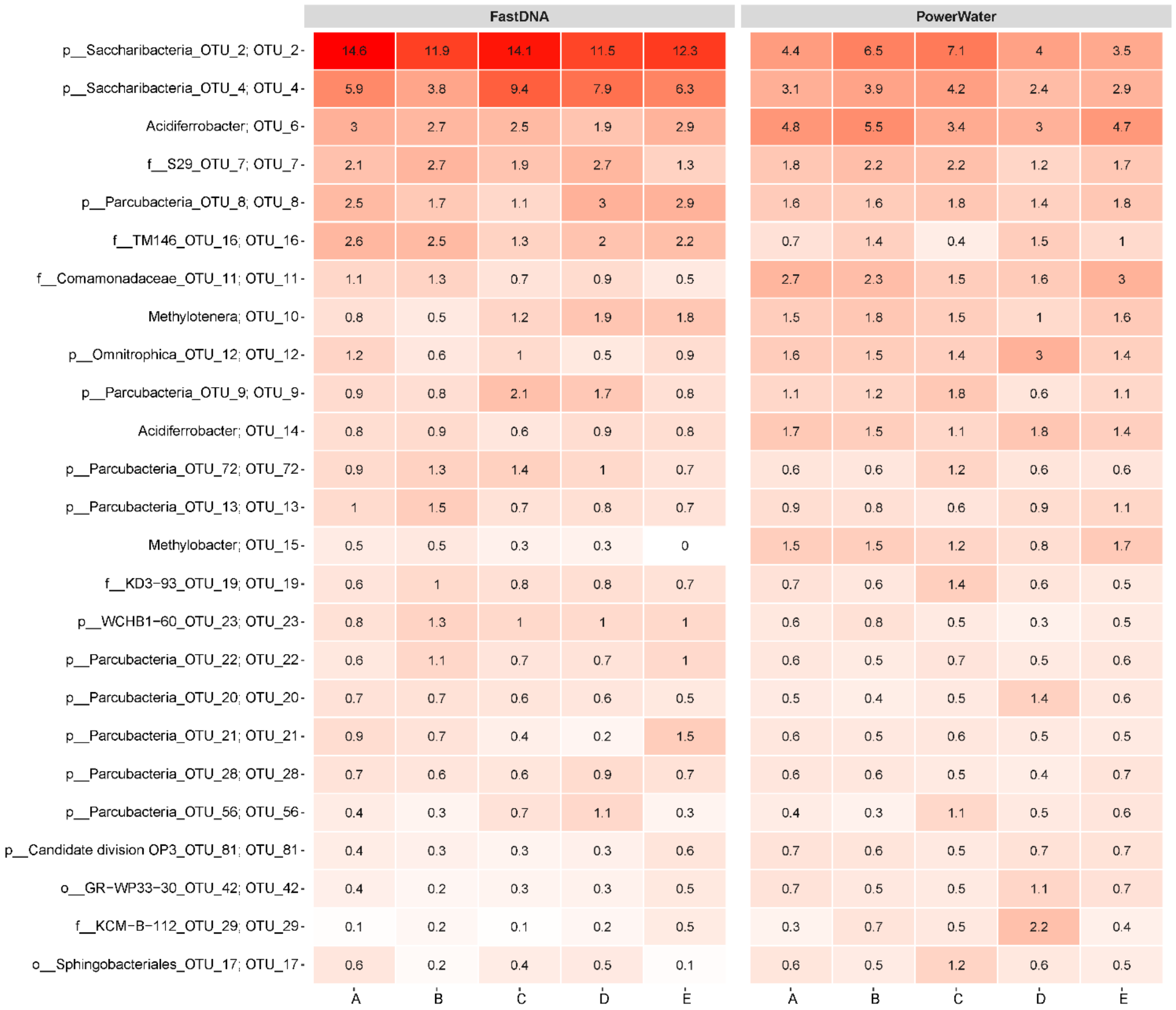


**Supplementary Figure 1.** Heatmap of 2 L drinking water samples extracted with two different kits. Each column represents a sample and is grouped by extraction kit. The rows list the 25 most abundant OTUs across the samples. Each OTU is assigned with its genus classification. The numbers state the relative read abundance.

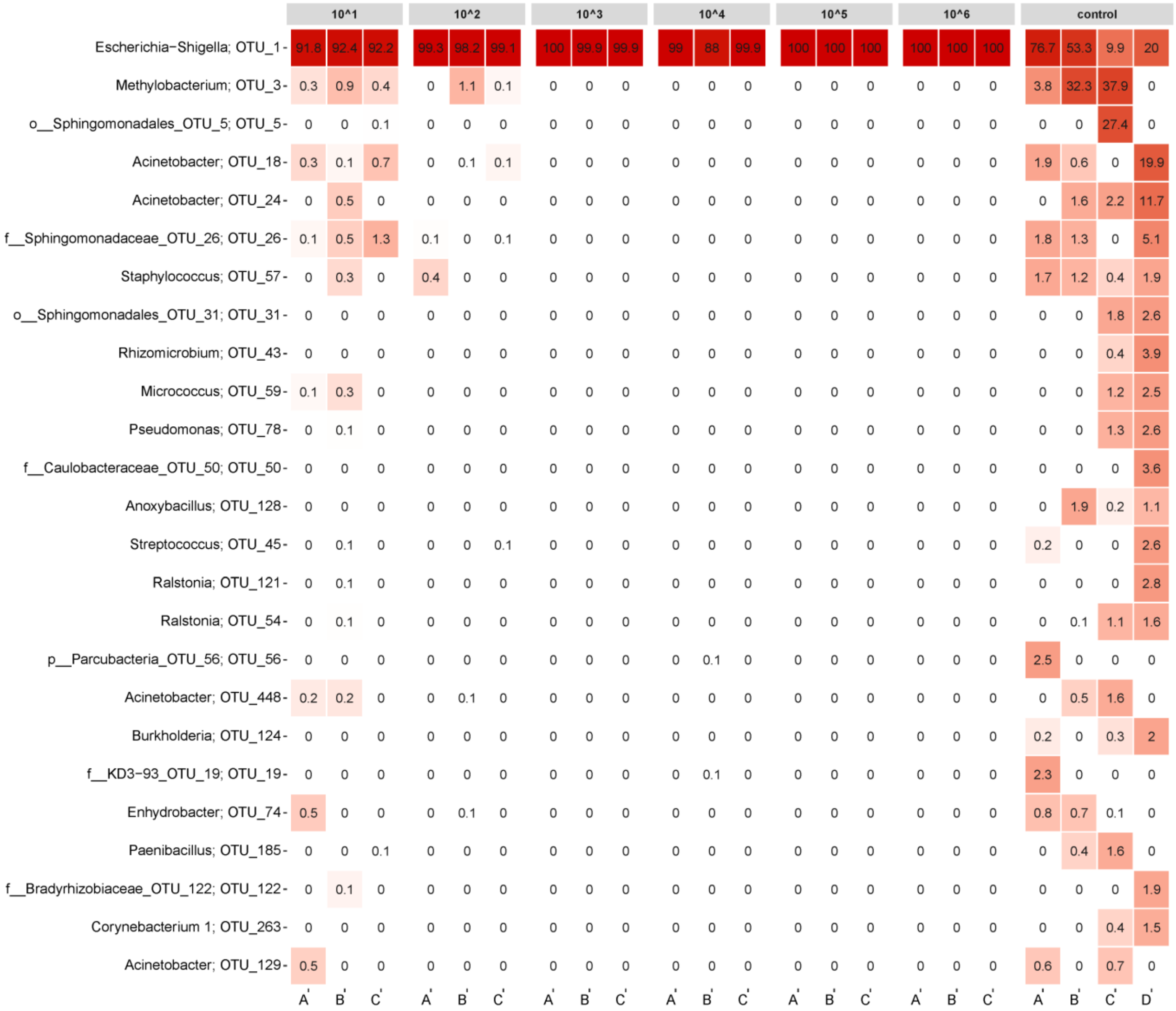

| | 10^1 | | | 10^2 | | | 10^3 | | | 10^4 | | | 10^5 | | | 10^6 | | | control | | | |
|---|---|---|---|---|---|---|---|---|---|---|---|---|---|---|---|---|---|---|---|---|---|---|
| | A | B | C | A | B | C | A | B | C | A | B | C | A | B | C | A | B | C | A | B | C | D |
| Escherichia−Shigella; OTU_1 | 91.8 | 92.4 | 92.2 | 99.3 | 98.2 | 99.1 | 100 | 99.9 | 99.9 | 99 | 88 | 99.9 | 100 | 100 | 100 | 100 | 100 | 100 | 76.7 | 53.3 | 9.9 | 20 |
| Methylobacterium; OTU_3 | 0.3 | 0.9 | 0.4 | 0 | 1.1 | 0.1 | 0 | 0 | 0 | 0 | 0 | 0 | 0 | 0 | 0 | 0 | 0 | 0 | 3.8 | 32.3 | 37.9 | 0 |
| o__Sphingomonadales_OTU_5; OTU_5 | 0 | 0 | 0.1 | 0 | 0 | 0 | 0 | 0 | 0 | 0 | 0 | 0 | 0 | 0 | 0 | 0 | 0 | 0 | 0 | 0 | 27.4 | 0 |
| Acinetobacter; OTU_18 | 0.3 | 0.1 | 0.7 | 0 | 0.1 | 0.1 | 0 | 0 | 0 | 0 | 0 | 0 | 0 | 0 | 0 | 0 | 0 | 0 | 1.9 | 0.6 | 0 | 19.9 |
| Acinetobacter; OTU_24 | 0 | 0.5 | 0 | 0 | 0 | 0 | 0 | 0 | 0 | 0 | 0 | 0 | 0 | 0 | 0 | 0 | 0 | 0 | 0 | 1.6 | 2.2 | 11.7 |
| f__Sphingomonadaceae_OTU_26; OTU_26 | 0.1 | 0.5 | 1.3 | 0.1 | 0 | 0.1 | 0 | 0 | 0 | 0 | 0 | 0 | 0 | 0 | 0 | 0 | 0 | 0 | 1.8 | 1.3 | 0 | 5.1 |
| Staphylococcus; OTU_57 | 0 | 0.3 | 0 | 0.4 | 0 | 0 | 0 | 0 | 0 | 0 | 0 | 0 | 0 | 0 | 0 | 0 | 0 | 0 | 1.7 | 1.2 | 0.4 | 1.9 |
| o__Sphingomonadales_OTU_31; OTU_31 | 0 | 0 | 0 | 0 | 0 | 0 | 0 | 0 | 0 | 0 | 0 | 0 | 0 | 0 | 0 | 0 | 0 | 0 | 0 | 0 | 1.8 | 2.6 |
| Rhizomicrobium; OTU_43 | 0 | 0 | 0 | 0 | 0 | 0 | 0 | 0 | 0 | 0 | 0 | 0 | 0 | 0 | 0 | 0 | 0 | 0 | 0 | 0 | 0.4 | 3.9 |
| Micrococcus; OTU_59 | 0.1 | 0.3 | 0 | 0 | 0 | 0 | 0 | 0 | 0 | 0 | 0 | 0 | 0 | 0 | 0 | 0 | 0 | 0 | 0 | 0 | 1.2 | 2.5 |
| Pseudomonas; OTU_78 | 0 | 0.1 | 0 | 0 | 0 | 0 | 0 | 0 | 0 | 0 | 0 | 0 | 0 | 0 | 0 | 0 | 0 | 0 | 0 | 0 | 1.3 | 2.6 |
| f__Caulobacteraceae_OTU_50; OTU_50 | 0 | 0 | 0 | 0 | 0 | 0 | 0 | 0 | 0 | 0 | 0 | 0 | 0 | 0 | 0 | 0 | 0 | 0 | 0 | 0 | 0 | 3.6 |
| Anoxybacillus; OTU_128 | 0 | 0 | 0 | 0 | 0 | 0 | 0 | 0 | 0 | 0 | 0 | 0 | 0 | 0 | 0 | 0 | 0 | 0 | 0 | 1.9 | 0.2 | 1.1 |
| Streptococcus; OTU_45 | 0 | 0.1 | 0 | 0 | 0 | 0.1 | 0 | 0 | 0 | 0 | 0 | 0 | 0 | 0 | 0 | 0 | 0 | 0 | 0.2 | 0 | 0 | 2.6 |
| Ralstonia; OTU_121 | 0 | 0.1 | 0 | 0 | 0 | 0 | 0 | 0 | 0 | 0 | 0 | 0 | 0 | 0 | 0 | 0 | 0 | 0 | 0 | 0 | 0 | 2.8 |
| Ralstonia; OTU_54 | 0 | 0.1 | 0 | 0 | 0 | 0 | 0 | 0 | 0 | 0 | 0 | 0 | 0 | 0 | 0 | 0 | 0 | 0 | 0 | 0.1 | 1.1 | 1.6 |
| p__Parcubacteria_OTU_56; OTU_56 | 0 | 0 | 0 | 0 | 0 | 0 | 0 | 0 | 0 | 0 | 0.1 | 0 | 0 | 0 | 0 | 0 | 0 | 0 | 2.5 | 0 | 0 | 0 |
| Acinetobacter; OTU_448 | 0.2 | 0.2 | 0 | 0 | 0.1 | 0 | 0 | 0 | 0 | 0 | 0 | 0 | 0 | 0 | 0 | 0 | 0 | 0 | 0 | 0.5 | 1.6 | 0 |
| Burkholderia; OTU_124 | 0 | 0 | 0 | 0 | 0 | 0 | 0 | 0 | 0 | 0 | 0 | 0 | 0 | 0 | 0 | 0 | 0 | 0 | 0.2 | 0 | 0.3 | 2 |
| f__KD3−93_OTU_19; OTU_19 | 0 | 0 | 0 | 0 | 0 | 0 | 0 | 0 | 0 | 0 | 0.1 | 0 | 0 | 0 | 0 | 0 | 0 | 0 | 2.3 | 0 | 0 | 0 |
| Enhydrobacter; OTU_74 | 0.5 | 0 | 0 | 0 | 0.1 | 0 | 0 | 0 | 0 | 0 | 0 | 0 | 0 | 0 | 0 | 0 | 0 | 0 | 0.8 | 0.7 | 0.1 | 0 |
| Paenibacillus; OTU_185 | 0 | 0 | 0.1 | 0 | 0 | 0 | 0 | 0 | 0 | 0 | 0 | 0 | 0 | 0 | 0 | 0 | 0 | 0 | 0 | 0.4 | 1.6 | 0 |
| f__Bradyrhizobiaceae_OTU_122; OTU_122 | 0 | 0.1 | 0 | 0 | 0 | 0 | 0 | 0 | 0 | 0 | 0 | 0 | 0 | 0 | 0 | 0 | 0 | 0 | 0 | 0 | 0 | 1.9 |
| Corynebacterium 1; OTU_263 | 0 | 0 | 0 | 0 | 0 | 0 | 0 | 0 | 0 | 0 | 0 | 0 | 0 | 0 | 0 | 0 | 0 | 0 | 0 | 0 | 0.4 | 1.5 |
| Acinetobacter; OTU_129 | 0.5 | 0 | 0 | 0 | 0 | 0 | 0 | 0 | 0 | 0 | 0 | 0 | 0 | 0 | 0 | 0 | 0 | 0 | 0.6 | 0 | 0.7 | 0 |

**Supplementary Figure 2.** Heatmap of the detection limit experiment including control samples. Each column represents a sample and is grouped by bacteria concentration. The rows list the 25 most abundant OTUs across the samples. Each OTU is assigned with its genus or the closest possible taxonomic rank. The numbers state the relative read abundance.
